# Study on Anti-Constipation Effects of *Hemerocallis citrina* Baroni through a Novel Strategy of Network Pharmacology Screening

**DOI:** 10.3390/ijms24054844

**Published:** 2023-03-02

**Authors:** Yuxuan Liang, Xiaoyi Wei, Rui Ren, Xuebin Zhang, Xiyao Tang, Jinglan Yang, Xiaoqun Wei, Riming Huang, Gary Hardiman, Yuanming Sun, Hong Wang

**Affiliations:** 1 Guangdong Provincial Key Laboratory of Food Quality and Safety, College of Food Science, South China Agricultural University, Guangzhou 510642, China; 2 The Institute for Global Food Security, School of Biological Sciences, Queen’s University Belfast, Belfast BT7 1NN, UK; 3 Guangdong Laboratory for Lingnan Modern Agriculture, Guangzhou 510642, China

**Keywords:** daylily, constipation, 16S rRNA, transcriptomes, network pharmacology

## Abstract

Daylily (*Hemerocallis citrina* Baroni) is an edible plant widely distributed worldwide, especially in Asia. It has traditionally been considered a potential anti-constipation vegetable. This study aimed to investigate the anti-constipation effects of daylily from the perspective of gastro-intestinal transit, defecation parameters, short-chain organic acids, gut microbiome, transcriptomes and network pharmacology. The results show that dried daylily (DHC) intake accelerated the defecation frequency of mice, while it did not significantly alter the levels of short-chain organic acids in the cecum. The 16S rRNA sequencing showed that DHC elevated the abundance of *Akkermansia*, *Bifidobacterium* and *Flavonifractor*, while it reduced the level of pathogens (such as *Helicobacter* and *Vibrio*). Furthermore, a transcriptomics analysis revealed 736 differentially expressed genes (DEGs) after DHC treatment, which are mainly enriched in the olfactory transduction pathway. The integration of transcriptomes and network pharmacology revealed seven overlapping targets (*Alb*, *Drd2*, *Igf2*, *Pon1*, *Tshr*, *Mc2r* and *Nalcn*). A qPCR analysis further showed that DHC reduced the expression of Alb, Pon1 and Cnr1 in the colon of constipated mice. Our findings provide a novel insight into the anti-constipation effects of DHC.

## 1. Introduction

Constipation is one of the common gastrointestinal symptoms, and different definitions of constipation lead to a range of reported incidences (from 1% to 80%) [1,2,3]. The occurrence of constipation is considered to be multifactorial [4], and it can lead to decreased quality of life and increased medical costs for people [5]. In particular, elderly people and women are more likely to be affected by constipation. Only approximately 25% of constipated people use medical treatments because of the adverse effects of some drugs [5,6,7], indicating safe and effective natural products for constipation relief are attractive.

Daylily (*Hemerocallis citrina* Baroni, HC) is an Asphodelaceae plant widely distributed around the world. It is cultivated as an ornamental species in Europe and North and South America [8], and thousands of cultivars have been registered [9]. The flowers of various *Hemerocallis* species have been used as an important vegetable in Asia, especially for *Hemerocallis citrina* and *Hemerocallis fulva* [10,11]. Among them, dried daylily (DHC) is a popular vegetable because of its delicious taste and various physiological activities. Some ancient medicine books, such as the *Compendium of Materia Medica* (*Ben Cao Gang Mu*), recorded that daylily can be used for anti-depression, promoting lactation, etc. These physiological activities of daylily have been reported in recent years [12,13,14]. *Ben Cao Fen Jing* (an ancient medicine book) also recorded the beneficial effects of daylily on the gut and stomach. In our previous research, we found more than 728 phytochemicals in daylily using UPLC-MS/MS, mainly including flavonoids, lipids, phenolic acids, amino acids and derivatives and organic acids [12,15]. Among them, flavonoids are one of the most abundant classes of compounds in daylily. The physiological activity of daylily polysaccharides was also reported recently [16]. It is well known that the benefits of flavonoids and polysaccharides on intestinal function have been widely reported [17,18,19,20]. However, the anti-constipation role of DHC is still unclear.

In this study, we investigated the anti-constipation effects of DHC using the defecation test and gastrointestinal transit test. Secondly, we aimed to evaluate the potential mechanism by measuring the content of short-chain organic acids (SCOAs) and the composition of gut microbiota in cecal contents. Then, we further evaluated the potential mechanism by integrating the network pharmacology and RNA sequencing. Lastly, we measured the expression of the related genes (*Alb*, *Pon1*, *Cnr1*, *Nos*, *Ache* and *Grp*).

## 2. Results

### 2.1. Anti-Constipation Effect of DHC

**Figure 1:**
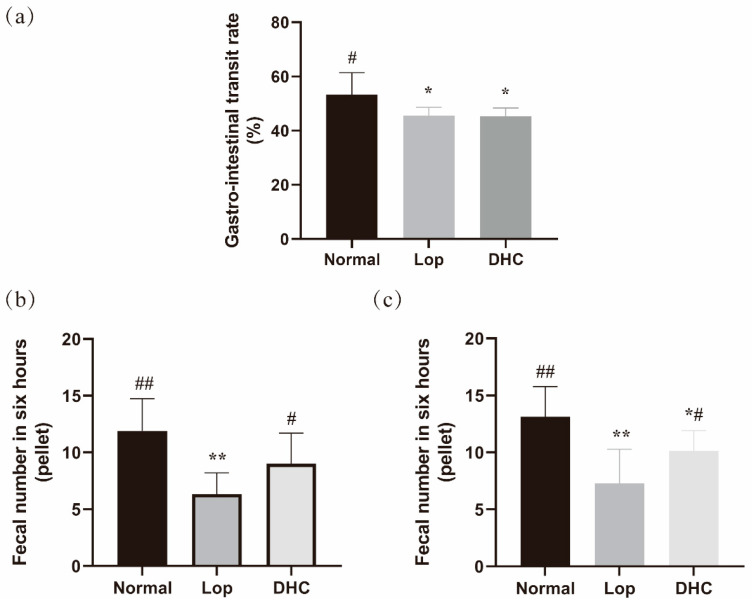
Gastrointestinal transit rate and defecation frequency in six hours of mice. Normal: distilled water-treated; Lop: Loperamide-treated; DHC: dry daylily-treated. (**a**) Gastrointestinal transit rate, (**b**) fecal number in six hours on day 13 and (**c**) fecal number in six hours on day 14; # represents the comparison with Lop group, *p* < 0.05; ## represents the comparison with Lop group, *p* < 0.01; * represents the comparison with Normal group, *p* < 0.05; ** represents the comparison with Normal group, *p* < 0.01.

Compared with the Normal group (distilled water), the gastrointestinal transit rate and defecation number of the loperamide (Lop) group were significantly regulated (*p* < 0.05), which indicated that the constipated model was built successfully (Figure 1). For the gastrointestinal transit test, DHC did not significantly accelerate the gastrointestinal transit rate in constipated mice (Figure 1a). However, for the defecation test, compared with the Lop group, DHC treatment significantly promoted the defecation number of constipated mice (Figure 1b,c, *p* < 0.05). These results suggest that the acceleration of large intestinal peristalsis can be responsible for the increased defecation frequency in constipated mice.

### 2.2. Effect of DHC on the Content of Short-Chain Organic Acids

**Figure 2:**
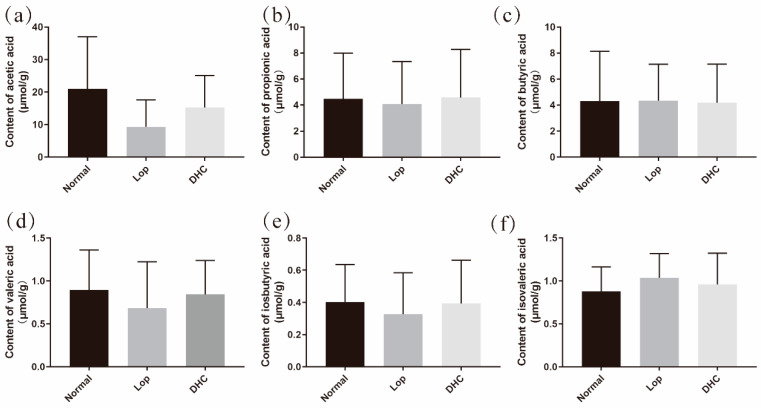
Short-chain organic acid content. Normal: distilled water-treated; Lop: Loperamide-treated; DHC: dry daylily-treated. (**a**–**f**) acetic acid, propionic acid, butyric acid, valeric acid, isobutyric acid and isovaleric acid.

The SCOAs of the cecal contents were further evaluated to test the impact of daylily on the intestinal environment in mice (Figure 2). There were no significant differences in the levels of acetic acid, propionic acid, butyric acid, isobutyric acid, valeric acid and isovaleric acid between the DHC group and the Lop group. However, compared with the Lop group, the administration of DHC elevated the contents of acetic acid (increased by 64.0%) and valeric acid (increased by 23.5%).

### 2.3. Effect of DHC on Gut Microbiota

**Figure 3:**
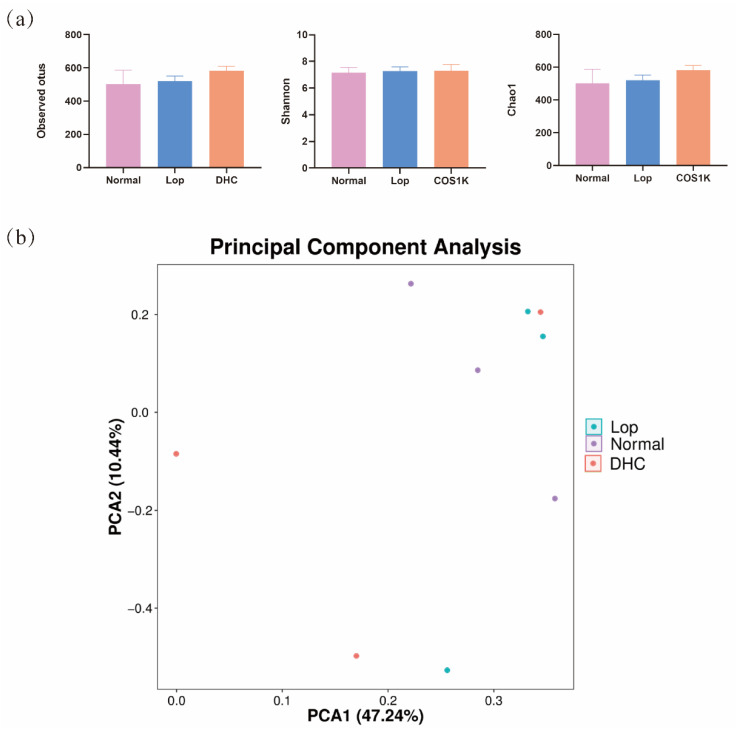
Alpha diversity and Beta diversity analysis. Normal: distilled water-treated; Lop: Loperamide-treated; DHC: dry daylily-treated. (**a**) Alpha diversity (Observed otus, Shannon and Chao1) and (**b**) Beta diversity (Principal component analysis).

**Figure 4:**
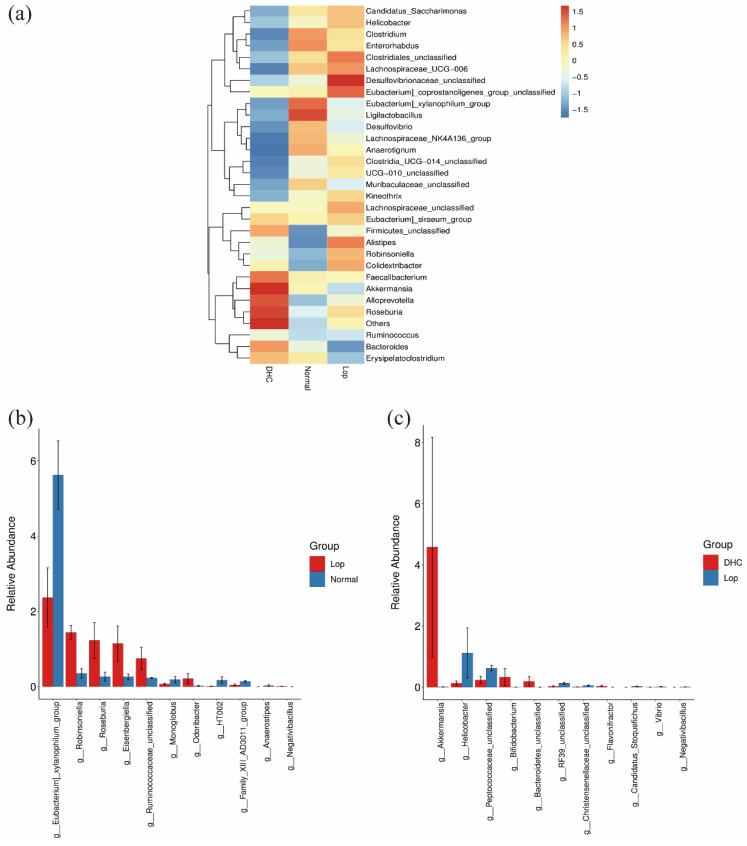
Intestinal flora composition analysis. Normal: distilled water-treated; Lop: Loperamide-treated; DHC: dry daylily-treated. (**a**) Relative abundance of the top 30 genera with a heatmap, (**b**) genera that changed significantly were between Lop and Normal and (**c**) genera that changed significantly were between DHC and Lop.

The 16S rRNA sequencing of the cecal contents was performed to characterize the effect of DHC on the intestinal flora. The results show that the administration of DHC did not significantly promote the alpha diversity of the intestinal flora (Figure 3a), whereas it affected the structure of the gut microbiota (Figure 3b). In addition, the relative abundance of the top 30 genera was further visualized with a heatmap (Figure 4a). To further reveal the difference in the community of gut microbiota, the relative abundance of the genus was analyzed. As shown in Figure 4b, the levels of *[Eubacterium]_xylanophilum_group*, *Monoglobus*, *Family_XIII_AD3011_group*, *HT002* and *Anaerostipes* were significantly reduced in the Lop group compared with those of the Normal group (*p* < 0.05). Conversely, the levels of *Robinsoniella*, *Roseburia*, *Eisenbergiella*, *Robinsoniella*, *Ruminococcaceae_unclassified*, *Odoribacter* and *Negativibacillus* were significantly increased in the Lop group than in those of the Normal group (*p* < 0.05). Compared with the Lop group, DHC promoted the levels of *Akkermansia*, *Bifidobacterium*, *Bacteroidetes_unclassified* and *Flavonifractor* while decreasing the levels of *Peptococcaceae_unclassified*, *Helicobacter*, *RF39_unclassified*, *Christensenellaceae_unclassified*, *Vibrio*, *Candidatus_Stoquefichus* and *Negativibacillus* in comparison with the Lop group (Figure 4c, *p* < 0.05).

### 2.4. Network Pharmacology Strategy

According to the target data of the current drug and disease database on constipation indications, a total of 309 constipation-related targets (*Homo sapiens*) were screened from the GeneCards, DrugBank and DisGeNET databases. After the conversion of species targets in the STRING database, a total of 278 constipation-related targets (*Mus musculus*) were obtained.

### 2.5. RNA Sequencing of Colon

**Figure 5:**
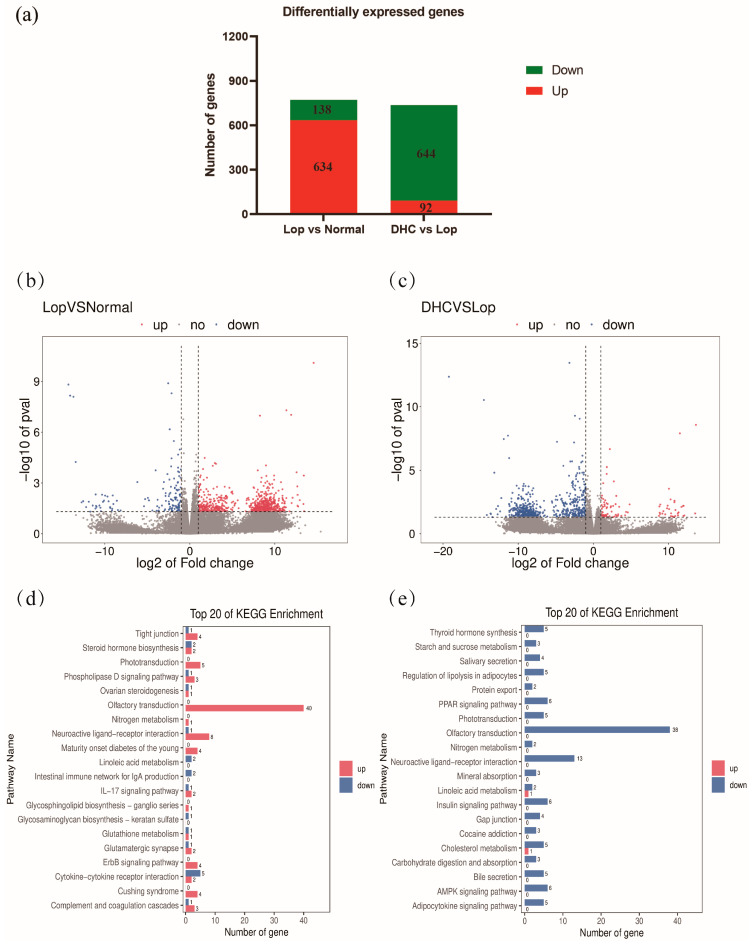
Transcriptomic analysis. (**a**) differential expression genes in different groups, (**b**) volcano plot of differential expression genes between Lop and Normal, (**c**) volcano plot of differential expression genes between DHC and Lop, (**d**) KEGG pathway enrichment of differential expression genes between Lop and Normal and (**e**) KEGG pathway enrichment of differential expression genes between DHC and Lop.

Transcriptome profiling of colon tissue was further used to investigate gene expression regulated by DHC. There are 772 differentially expressed genes (DEGs) found between the Lop group and the Normal group (Figure 5a,b and Table S2). Compared with the Normal group, a total of 634 genes were up-regulated in the Lop group, while 138 genes were down-regulated in the Lop group. In addition, a total of 736 DEGs were observed between the DHC group and the Lop group (Figure 5a,c and Table S3). Compared with the Lop group, a total of 92 genes were up-regulated, whereas 644 genes were down-regulated in the Lop group.

### 2.6. KEGG Pathway Enrichment

DEGs between groups were further used to perform KEGG functional enrichment. As a result, the top 10 KEGG pathways between the Lop group and the Normal group were Olfactory transduction, Phototransduction, Maturity onset diabetes of the young, ErbB signaling pathway, Complement and coagulation cascades, Steroid hormone biosynthesis, Neuroactive ligand–receptor interaction, Tight junction, Intestinal immune network for IgA production and Cytokine–cytokine receptor interaction (Figure 5d, *p* < 0.05). In addition, the top 10 KEGG pathways between the DHC group and the Lop group were Olfactory transduction, Phototransduction, Cholesterol metabolism, Regulation of lipolysis in adipocytes, PPAR signaling pathway, Neuroactive ligand–receptor interaction, Adipocytokine signaling pathway, Bile secretion, Thyroid hormone synthesis and Starch and sucrose metabolism (Figure 5e, *p* < 0.05).

### 2.7. Joint Analysis of Network Pharmacology and RNA Sequencing

**Figure 6:**
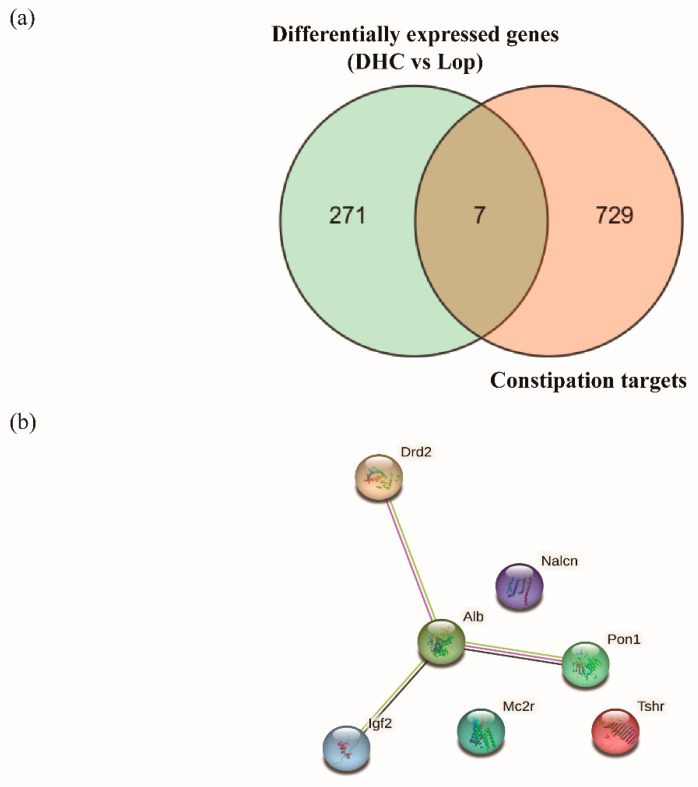
Joint analysis of network pharmacology and transcriptomes. (**a**) Venn diagram for the differential expression genes (Lop vs. Normal) and constipation targets and (**b**) Protein–protein interaction network of overlapping targets.

To further investigate the relationship between DEGs and constipation targets, a Venn diagram was used to illustrate the overlapping targets between the DEGs (DHC vs. Lop) and constipation targets. The results show that seven overlapping targets were found (Figure 6a). To further understand the relationship among these seven targets (*Alb*, *Drd2*, *Igf2*, *Pon1*, *Tshr*, *Mc2r* and *Nalcn*), the PPI relationships of these seven targets were further displayed using the PPI networks (Figure 6b and Table 1). The results show that Alb and Pon1 were the most closely associated targets in the PPI network.

### 2.8. mRNA Expression Analysis

**Figure 7:**
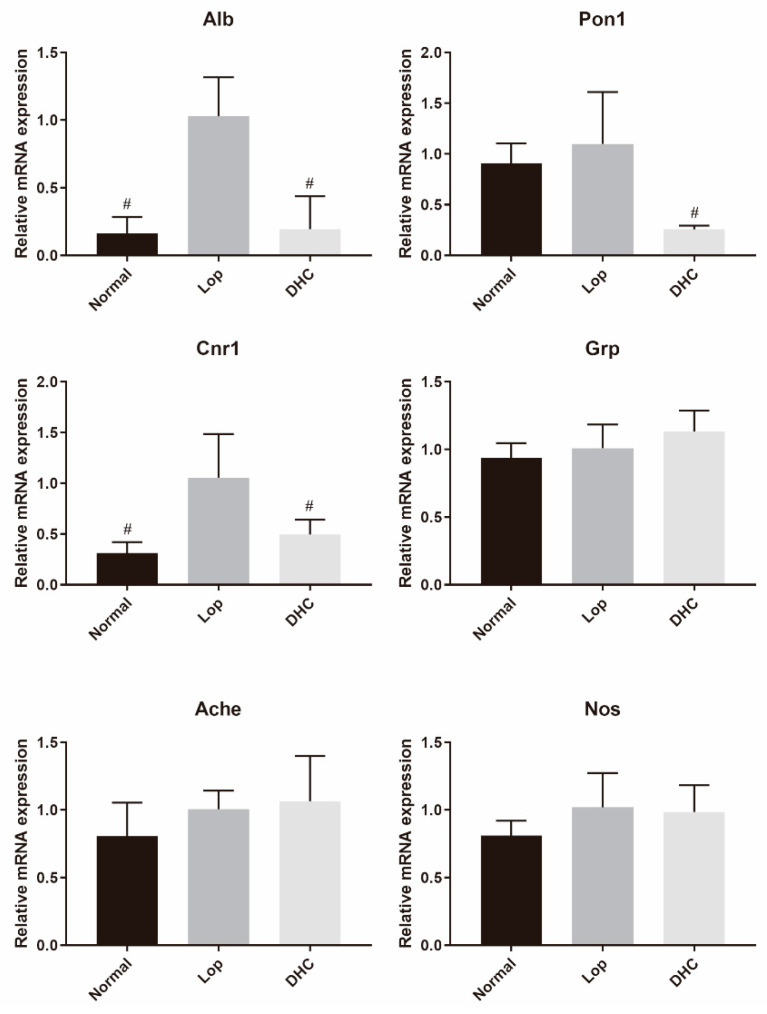
Relative expression of genes (mean ± SD). Normal: distilled water-treated; Lop: Loperamide-treated; DHC: dry daylily-treated; # represents the comparison with Lop group, *p* < 0.05.

The relative mRNA expression of the core targets (*Alb* and *Pon1*) from the PPI network and other constipation-related targets (*Cnr1*, *Nos*, *Ache* and *Grp*) were further analyzed by qPCR. As a result, compared with the Lop group, DHC treatment significantly reduced the relative expression of *Alb*, *Cnr1* and *Pon1* in constipated mice (Figure 7, *p* < 0.05).

## 3. Discussion

Daylily is a food resource that has a long history of consumption in Asia given its delicious taste and various physiological activities. Previous studies have shown that daylily contains many potential anti-constipation components, such as flavonoids and polysaccharides. In this study, the defecation frequency and gastrointestinal transit were firstly adopted to investigate the anti-constipation effect of DHC. Then, the SCOAs and 16s rRNA sequencing of cecal contents were further performed to investigate the anti-constipation effects of DHC. Lastly, the perspective of the transcriptomes and network pharmacology was adopted to elucidate the underlying mechanisms of DHC.

Constipation is a common symptom affecting people of all ages, and it results in an expensive burden on the economy [21]. Although laxative drugs are used to treat constipation and have good effects, side effects have been reported with using these drugs [22]. Recently, dietary supplements (such as natural products, prebiotics and probiotics) with anti-constipation effects have drawn the attention of researchers due to their good effectiveness, high safety and low costs [23,24,25]. In this study, DHC treatment did not promote the gastrointestinal transit but significantly accelerated the defecation frequency of constipated mice. 

The gut metabolites (SCOAs) are closely related to the development of constipation [26,27]. To investigate the anti-constipation role of DHC, the contents of SCOAs in the cecal contents were further measured by GC. As a result, although increasing trends of acetic acid and valeric acid were found, the contents of SCOAs (acetic acid, propionic acid, butyric acid, isobutyric acid, valeric acid and isovaleric acid) were not significantly regulated by DHC. These results suggest that the constipation-relieving effects of DHC may not involve the regulation of SCOAs in the cecum.

Accumulating evidence reported that alterations in the intestinal microbiota of the host are closely associated with the regulation of constipation. In this study, 16S rRNA sequencing was performed to characterize the regulation of DHC on the intestinal flora in constipated mice. The results show that 11 genera were significantly regulated (*p* < 0.05). *Bifidobacterium* is a well-known intestinal probiotic, and accumulating evidence suggested that increased *Bifidobacterium* was beneficial for constipation relief [28,29]. Our results show that DHC significantly promoted the levels of *Bifidobacterium* (*p* < 0.05). *Akkermansia* reportedly plays a positive role in metabolic modulation and gut health protection [30,31,32,33]. For example, *Akkermansia* can decrease the pro-inflammatory factor expression to relieve ulcerative colitis [34]. Previous studies reported that some probiotics (Bifidobacterium longum and Lactobacillus plantarum KFY02) and symbiotics can alleviate constipation, and they all promoted the relative abundance of *Akkermansia* [32,35,36]. In this study, DHC elevated the levels of *Akkermansia* (*p* < 0.05). *Flavonifractor* is a flavonoid-degrading bacterium [37], and our results show that DHC promoted the level of increased *Flavonifractor*, suggesting gut microbiota can utilize flavonoids of DHC to exert a physiological effect. Taken together, these results reveal the anti-constipation effects of DHC involving the proliferation of beneficial bacteria and flavonoid-utilizing bacteria and the inhibition of harmful bacteria.

Furthermore, the KEGG pathway enrichment was performed to further reveal the underlying mechanism of DHC in constipation relief. In this study, the KEGG pathway enrichment of DEGs (Lop vs. Normal; DHC vs. Lop) showed that olfactory transduction was the most significantly enriched pathway. In this pathway, LOP up-regulated the expression of 40 genes compared with Normal, whereas DHC down-regulated the expression of 38 genes compared with Lop (Figure 5d,e). It is well known that SCOAs, indoles and ammonia are known to be odorous compounds in feces. SCOAs are considered beneficial to health, while indole and ammonia are the opposite. Feces odor is associated with constipation [38]. Protein catabolism in the gut may produce compounds that are toxic to the host, such as amines and indoles, which can potentially affect intestinal motility [39,40,41]. A previous study reported that lactosucrose treatment significantly reduced the concentrations of p-cresol, indole, skatole and ammonia in feces in the elderly with constipation [42]. We speculate that some harmful odorous compounds are produced in the gut of constipated mice, while DHC treatment reduces the level of these odorous compounds. These results indicate that olfactory transduction is closely related to the anti-constipation role of DHC. However, the role of olfactory transduction in the anti-constipation of DHC still needs further study.

In recent years, the network pharmacology method has emerged as an effective strategy for establishing relationships between genes and diseases [43,44]. Herein, we adopted the network pharmacology strategy to systematically collect constipation-related targets through GeneCards, DrugBank and DisGeNET. According to the list of overlapping targets, the indirect relationship between DEGs (DHC vs. Lop) and constipation targets was established. As a result, we found that DEGs (DHC vs. Lop) and constipation targets shared seven overlapping targets, and the PPI network of overlapping targets further revealed that *Alb* and *Pon1* were the two main targets in the PPI network. *Alb* encodes serum albumin, and the constipation scoring system was significantly and negatively correlated with the serum albumin level [45,46]. Furthermore, *Alb* was regarded as a core anti-constipation target of raffino-oligosaccharide [47]. *Pon1* was found to have been significantly related to chronic constipation in a cross-sectional study [48]. In this study, the administration of DHC significantly down-regulated the expression of *Alb* and *Pon1* in constipated mice (*p* < 0.05).

*Grp* codes a gastrin-releasing peptide, which is associated with bowel motility [49,50]. *Ache* and *Nos* are important regulators of gut peristalsis, and they are also common genes of interest in gastrointestinal motility studies [51,52,53]. However, in this study, the expression of these genes was not significantly regulated by DHC intervention. A previous study reported that the activation of *Cnr1* receptors slowed down the peristalsis of the colon [54], while a *Cnr1* inverse agonist relieved the slow gastrointestinal motility [55]. Herein, DHC treatment significantly down-regulated the expression of *Cnr1* (Figure 7, *p* < 0.05). In a word, the qPCR analysis suggested that the anti-constipation effect of DHC involved the regulation of *Alb*, *Pon1* and *Cnr1*.

## 4. Materials and Methods

### 4.1. Materials

The dried daylily (flower bud) was obtained from Yunxing Lake Modern Agricultural Center (Qidong, China), and it was subjected to superfine grinding according to the research of Hu et al. [56]. The loperamide hydrochloride was purchased from Dashenlin Pharmacy, which was produced in Janssen Pharmaceutical Ltd. (Xi’an, China). The active charcoal and gum Arabic were bought from Hengxing (Tianjin, China).

### 4.2. Mice

A total of 30 male BALB/c mice (7 weeks old and weighing 20 g ± 2 g) were purchased from Guangdong Medical Laboratory Animal Centre (Guangzhou, China). All mice were fed under standard conditions, and the Ethics Committee of South China Agricultural University (SYXK 2019-0136) approved this experiment.

### 4.3. Animal Experiment Design

For defecation test, 30 mice were randomly divided into three groups (n = 10) after 7 days of adaptive feeding: normal control group (Normal, distilled water), constipation model group (Lop, distilled water) and dried daylily group (DHC, DHC suspension). Three groups were administered with 0.5 mL/mouse/day in the corresponding sample by vialing gavage once a day for 14 days. On this basis, 30 min after completing the original gavage, the Lop group and the DHC group were treated with loperamide (5 mg/kg body weight; 0.5 mL) via gavage from Day 12 to Day 14 to induce constipation [29,57]. Correspondingly, the Normal group was given an additional 0.5mL distilled water from Day 12 to Day 14. Then, the defecation status (fecal numbers in six hours) of each mouse in a separate cage was observed. DHC powder and Lop powder were distributed in distilled water as a suspension. A 10 g/day dosage of dried daylily for an adult (70 kg) was considered, which is equivalent to 0.14 g/kg per day. Referring to the technical standards for testing and assessment of functional food formulated by China Food and Drug Administration and our previous research [57], mice were administered with DHC at a dose of 1.4 g/kg body weight/day.

All groups fasted for 16 h (water was available) before measurement of the gastro-intestinal transit. At the end of the experiment, after giving Lop for 30 min, each group was intragastrically given activated carbon suspension (containing 5% activated carbon and 10% gum Arabic) containing corresponding samples (Normal group: water; Lop group: Lop; DHC group: DHC). After 25 min, the mice were anesthetized with pentobarbital and euthanized. The gastrointestinal transit rate was calculated using our previous method [57]. The cecal content and colon tissue were collected and stored at −80 °C.

### 4.4. Short-Chain Organic Acid Determination

The cecal contents of acetic acid, propionic acid, butyric acid, isobutyric acid, valeric acid and isovaleric acid in mice were detected by gas chromatography (GC, 7890B, Agilent, Santa Clara, CA, USA), and the corresponding standard samples were obtained from Macklin (Shanghai, China). For GC detection, FFAP elastic quartz capillary column (30 m × 0.25 mm × 0.25 μm) was used, and the initial temperature was 70 °C, then increased at 5 °C/min to 150 °C (maintained 2 min). Nitrogen was used as the carrier gas (flow rate 2 mL/min). The detector temperature was 280 °C, and the injection volume was 2 μL.

### 4.5. 16S rRNA Sequencing

The cecal contents of mice were used to perform 16S rRNA sequencing. Cetyltrimethylammonium bromide was used to extract DNA from the cecal content. The primers 341F (5′-CCTACGGGNGGCWGCAG-3′) and 805R (5′-GACTACHVGGGTATCTAATCC-3′) were used to amplify the V3–V4 variable region of 16S rRNA gene by PCR. The paired-end sequenced (2 × 250) was performed on the NovaSeq PE250 platform. Detailed information on the sequencing procedures was shown in the previous study [57].

### 4.6. Network Pharmacology Strategy

The constipation-related therapeutic targets were screened by GeneCards (four times the score of all the targets, the score > 3.79) [58], DrugBank [59] and DisGeNET 7.0 (the score > 0.1) [60]. To reveal the relationship between daylily and constipation, a Venn diagram was used to illustrate the overlapping targets between DHC targets and constipation targets. Protein–protein interaction (PPI) networks of the overlapping targets were constructed by STRING.

### 4.7. Transcriptomic Analysis

The proximal colons of the mice were cleaned with saline and stored in a −80 °C freezer until transcriptomic sequencing. The total RNA of these tissues was isolated and purified with the TRIzon kit (CWBIO, Beijing, China) and the RNA was reverse transcribed according to the manufacturer’s instructions. (Invitrogen, Carlsbad, CA, USA). Quantity and purity of total RNA were evaluated by NanoDrop ND-1000 (NanoDrop, Wilmington, DE, USA), and the integrity of RNA was detected by Bioanalyzer 2100 (Agilent, Santa Clara, CA, USA). RNA libraries were created using the TruSeq RNA sample preparation kit (Illumina, San Diego, CA, USA). Illumina NovaseqTM 6000 was used for the RNA sequencing, and the read length of PE150 was adopted. The details of the transcriptomic analysis were consistent with the previous study [61]. For analysis of differential expression, the screening criteria of DEGs were set as FC ≥ 2 or FC ≤ 0.5 and *p*-value < 0.05.

### 4.8. Quantitative Real-Time PCR

The RNA samples for qPCR analysis were selected from the same colon tissues used for RNA sequencing. qPCR analysis was performed using a TB Green^®^ Premix Ex Taq™ II kit (Takara, Shanghai, China) and Bio-Rad C1000 Thermal Cycler Real-Time PCR System (Bio-Rad, Hercules, CA, USA). The reverse transcription reaction system is a final volume of 10 μL, including 1 μg RNA, 1 μL PrimeScript RT Enzyme MixⅠ, 1 μL RT Primer Mix, 4 μL 5 × PrimeScript Buffer and RNase-free water (37 °C for 15 min and then 85 °C for 5 s). Amplification volume was 20 μL containing 2 μL cDNA, 0.8 μL forward primer (10 μM), 0.8 μL reverse primer (10 μM), 0.4 μL ROX Reference Dye (50×), 10 μL SYBR Premix Ex Taq Ⅱ and 6 μL RNase free water. The amplification conditions were a pre-denaturation program (95 °C for 30 s), and the amplification program (95 °C for 5 s, and 60 °C for 34 s) was for 40 cycles. The expression level of *Gapdh* was normalized [12]. Table S1 provides detailed information on the primers used. The relative expression levels of gene expression were calculated by the ΔΔCt method.

### 4.9. Statistical Analysis

The Least Significant Difference Test and Kruskal–Wallis Test (SPSS version 20.0) were used to analyze the differences between groups according to whether the variances were consistent between groups [57,62]. All data were expressed with the mean ± SD, and a *p* < 0.05 was considered statistically significant.

## 5. Conclusions

Our findings reveal that the administration of DHC accelerated the defecation frequency of mice. It elevated the abundance of *Akkermansia*, *Bifidobacterium* and *Flavonifractor* in cecal contents while reducing the levels of pathogens (such as *Helicobacter* and *Vibrio*) in cecal contents. A transcriptomic analysis further found 736 DEGs in the colon after DHC intervention, which mainly involved the olfactory transduction pathway. Furthermore, the integration of transcriptomes and network pharmacology revealed seven overlapping targets (*Alb*, *Drd2*, *Igf2*, *Pon1*, *Tshr*, *Mc2r* and *Nalcn*). A qPCR analysis further showed that DHC effectively down-regulated the expression of *Alb*, *Pon1* and *Cnr1* in the colon. These results improve the understanding of the anti-constipation effect of daylily and provide a novel integrated perspective of transcriptomes and network pharmacology.

## Figures and Tables

**Table 1:** Protein–protein interaction network.

Node 1	Node 2	Combined Score
*Alb*	*Pon1*	0.614
*Alb*	*Igf2*	0.573
*Alb*	*Drd2*	0.466

## Data Availability

The data in the current study are available from the corresponding author on reasonable request.

## Supplementary Materials

The following supporting information can be downloaded at: https://www.mdpi.com/article/10.3390/ijms24054844/s1.
